# Effects of soil particle size on rainfall-induced erosion of near-horizontal layered slopes

**DOI:** 10.1371/journal.pone.0331153

**Published:** 2025-09-22

**Authors:** Chenxin Liu, Zizhao Zhang, Lilong Cheng, Yamei Wang, Xinyu Liu, Runsen Lai, Qianli Lyv

**Affiliations:** 1 School of Geology and Mining Engineering, Xinjiang University, Urumqi, China; 2 Changji Geological Brigade, Geological Bureau, Changji, XinJiang Uyghur Autonomous Region, China; 3 Research Base of Xinjiang University, State Key Laboratory of Deep Rock Mechanics and Underground Engineering, Urumqi, China; 4 China University of Geosciences, Beijing, China; 5 China University of Mining and Technology, Xuzhou, Jiangsu, China; Guizhou University, CHINA

## Abstract

The problem of soil erosion is prominent in the arid region of northwest China, and the Kapo in Wensu County, Xinjiang is highly susceptible to erosion and damage disasters due to the formation of chalky high steep slopes affected by rivers. In order to provide theoretical support for slope prevention and control, this paper investigates the erosion damage characteristics of three different grain size compositions of nearly horizontal laminated soil slopes under rainfall by using rainfall simulation equipment in an indoor model box test. The results show that rainfall, slope soil properties and cracks are the main causes of erosion damage on slopes. As rainfall increases, the slope shows regressive damage from the foot of the slope to the top of the slope. Soil slopes with coarse-grained sand have less erosion damage due to good permeability of the sand layer; slopes with fine soil particles have weak erosion resistance and severe gully erosion. The dominant seepage channels formed by cracks play a significant role in the erosion damage of slopes.

## 1. Introduction

In the north and east of Wensu county town in the arid region of Xinjiang, northwest China, under the erosive action of the Kumarik River, the front edge of the slopes is severely cut, the slope gradient is almost upright, the upper vertical joints, cracks as well as fall holes are developed, and the characteristics of the slopes are remarkable, and coupled with the unique climatic and geomorphological environments, soil erosion is the most serious problem faced at present [1,2]. Slope erosion is mainly influenced by rainfall intensity, soil particle size, crack development, slope gradient and other factors [3–5]. Soil erosion in the region has been accelerated by the continuous development of urban construction, the increased frequency of human engineering and economic activities, and the influence of rainfall, snowmelt, earthquakes and other triggering factors, and unstable slopes exist in a number of places, seriously threatening the safety of residents’ lives and property. An in-depth study of soil erosion in the region is of great importance to ensure the safety of residents and to promote the sustainable development of the region.

Scholars have conducted an in-depth study on the influencing factors and formation mechanism of slope erosion, and concluded that depth and pore pressure have a significant effect on clay sensitivity parameters [6,7]. The physical properties of the soil control the degree of erosion of slopes, and the increase in pore pressure in the soil reduces the internal stresses in the soil, which reduces the strength of the soil [8,9], Wenshou Kapo is dominated by chalky soil layer with thin sand layer, the strength of chalky soil layer is low, cohesion is poor, experiencing long-term deposition, weathering, joints and cracks are developed, and the strength of the soil body rapidly decreases after rainfall infiltration to form gully erosion [10,11]. The degree of soil erosion is strongly related to the grain size composition of the soil body [12–14], and under rainfall conditions it was found that coarse sand affects the infiltration rate and the infiltration path of the soil. With more coarse particles, the internal porosity of the soil increases, which has a greater ability to promote infiltration and rainwater infiltration, but has a poorer water storage capacity. Coarse sand can effectively resist the impact of raindrops, causing small displacements and slowing the rate of soil erosion. A different particle size composition not only changes the internal structure of the soil, but also alters the internal hydromechanical properties, thus affecting the soil erosion process [15–17]. It was found that the absence of vegetation cover on the soil surface resulted in the susceptibility of soil slopes to erosion [18,19]. The degree of soil erosion is related to rainfall intensity [20], rainfall amount [21] and rainfall duration [22]. By studying the occurrence and evolution of gully erosion under rainfall conditions, it was found that rainfall intensity, rainfall amount and rainfall duration affect the infiltration of rainwater at the soil surface, resulting in an increase in slope runoff and severe soil erosion. Rainwater infiltration causes changes in the internal structure [23,24], reducing the stability of the soil [25], increasing the internal water content of the soil, increasing the pore water pressure, increasing the horizontal earth pressure and reducing the shear strength. The gradient of the slope determines the degree of erosion [26,27]. The more gentle the slope, the higher the rainfall infiltration rate, the infiltration depth and the infiltration rate at the top and toe of the slope are faster than in the middle of the slope, and the greater the area of soil erosion damage.

To sum up, in recent years, scholars for granite residual soil, loess slope erosion mechanism, factors affecting more research, but for similar Wensu County “Kapo” near horizontal chalk – fine sand interbedded soil characteristics of the slope research is less. The innovation of this paper lies in the study of the erosion evolution process and damage mechanism of soil slopes with different grain size compositions under rainfall conditions through an indoor model box test using rainfall simulation equipment [28,29]. The test can visualise the whole process of erosion and damage of soil slopes, monitor the changes of internal water content, pore water pressure and horizontal soil pressure during rainfall infiltration, analyse the characteristics of soil parameters in response to rainfall infiltration, explore the effects of rainfall on the stress and seepage fields of nearly horizontal layered soil slopes with different grain size compositions, and reveal the mode and mechanism of erosion and damage of soil slopes.

## 2. Raw materials and methodology

### 2.1. Sampling location

This study area is located in Wensu County, Aksu Region, Xinjiang Uygur Autonomous Region. It is a high and steep soil slope formed by the Kokyar River in the northeast and the Kumarik River in the southwest, and belongs to the pre-mountain river alluvial plain at the southern foot of the Tomur Peak, which is relatively flat in overall topography. As the Kumarik River continues to erode the edge of the Kokyar River alluvial plain, the junction of the two rivers’ alluvial plains has formed a steep slope of more than 10 kilometers in length, locally known as “Kapo”. The overall plan form of the Kapo is in the form of an inverted L shape, with a total area of approximately 20.26 km2. The overall height gradually decreases from north to south, the angle of which is about 7‰. Within this region, the lithology generally consists of the silty soil layers interspersed with thin layers of fine sand and gravelly sand. As a result, the dual-layer or multilayer structure with complex river sediment rhythms becomes to be the main feature of the lithology. Under the joint erosion of heavy rainfall or floods from the Kokyar River, there are numerous vertical slope gullies distributed along the Kapo and the length of which ranges from hundreds of meters to several kilometers.

### 2.2. Raw soil mass

**Fig 1:**
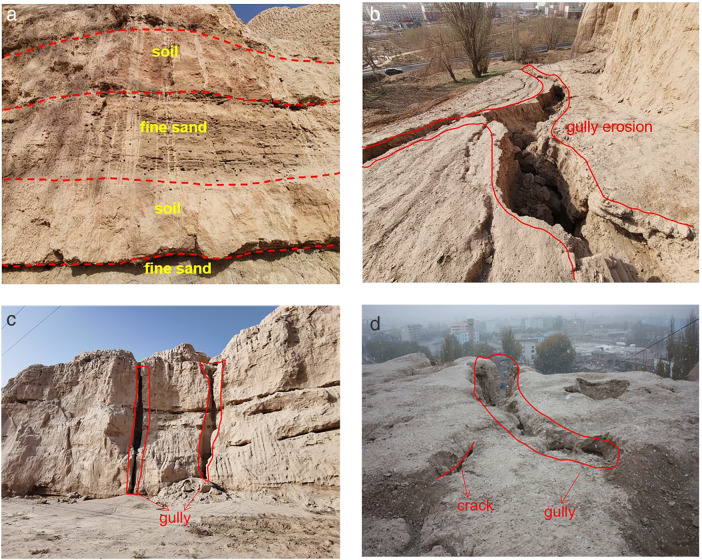
Stratigraphic lithology typical failures modes of the soil slope of the Kapo. (a) Vertical rhythmic layer. (b) Slope gully erosion. (c) Penetrated gully. (d) Slope cracks and gully.

**Fig 2:**
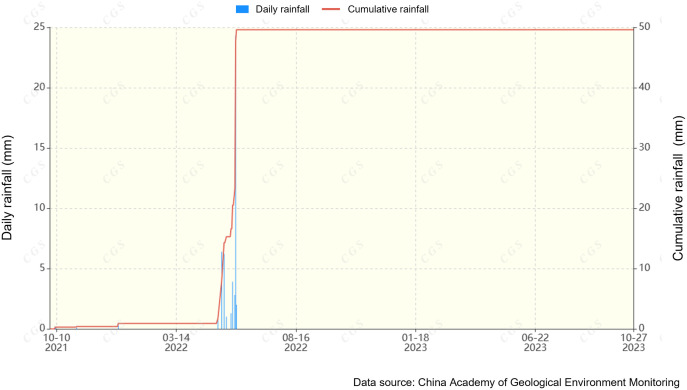
History data about the daily and cumulative rainfall.

The raw soil mass was collected from the northern slope of the Kapo in Wensu County. As can be seen from Fig 1, (Typical failures modes of the soil slope of the Kapo: (a)Vertical rhythmic layer; (b)Slope gully erosion; (c)Penetrated gully; (d)Slope cracks and gully), vertical layers of silty soil interspersed with sand layers are obvious and the severe erosion was existed at the top of the slope. The sampling site is a typical temperate continental climate with little rainfall and the highest temperatures of which is up to 40.9°C. Based on the rainfall gauge data shown in Fig 2, the short-duration heavy rainfall. The maximum rainfall of 69.1 mm in a single day was recorded at the monitoring station in the theatre study area on 16 May 2023, resulting in severe erosion of the slopes and the occurrence of a landslide hazard. This maximum rainfall was used as the basis for designing the rainfall erosion test.

**Fig 3:**
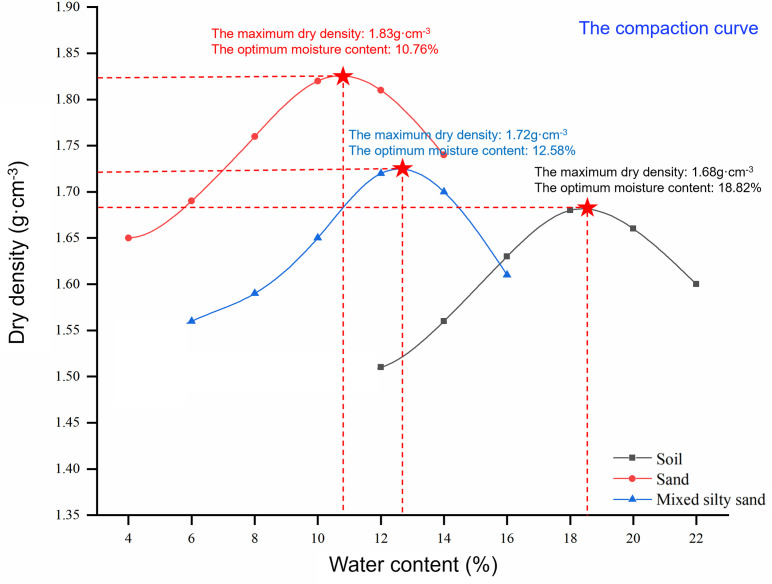
Compaction curve.

To maintain the similarity between the physical simulation model and the actual soil slope in practice, the undisturbed soil collected onsite with a depth of 1 meter below the sampling point were immediately sealed with plastic wrap and tested at the laboratory. Lots of physical and mechanical indicators were tested [30], including the moisture content, liquid limit, plastic limit, internal friction angle, cohesion, specific gravity, and maximum dry density. Three sets of control tests were carried out on each sample, for a total of nine tests. The mean values of the test results are given in the table below, (Tables 1–3) and the compaction curves were plotted in Fig 3.

**Table 1:** Physical properties of the raw soil mass for tests.

Dry Density	Water content	Density	Liquid limit	Plastic limit	Angle of internal friction	Cohesion
ρ/(g·cm-^3^)	W%	ρ/(g·cm-^3^)	WL%	WP%	φ°	kPa
1.96	18.82	1.68	21.6	15.2	33	11.7

**Table 2:** Physical properties of the fine sand for tests.

Dry Density	Water content	Density	Liquid limit	Plastic limit	Angle of internal friction	Cohesion
ρ/(g·cm^-3^)	W%	ρ/(g·cm^-3^)	W_L_%	W_P_%	φ°	kPa
1.83	10.76	2.11	25.7	18.3	21	10.6

**Table 3:** Physical properties of the mixed silty sand for tests.

Dry Density	Water content	Density	Liquid limit	Plastic limit	Angle of internal friction	Cohesion
ρ/(g·cm^-3^)	W%	ρ/(g·cm^-3^)	W_L_%	W_P_%	φ°	kPa
1.72	12.58	1.93	23.3	17.0	24	11.2

**Fig 4:**
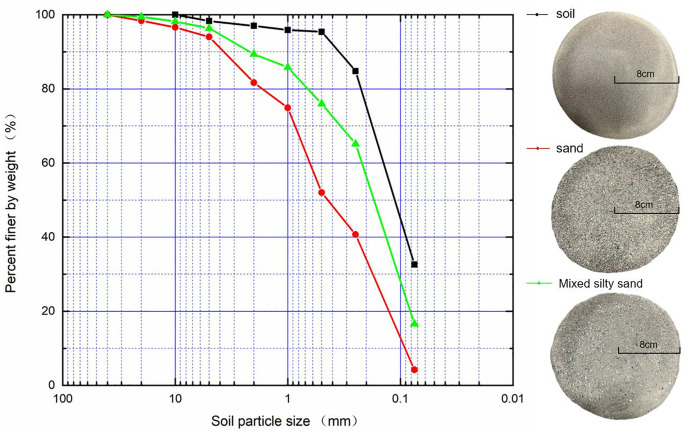
Particle size distribution of three typical soils.

To explore the effects of particle size on the rainfall erosion characteristics of slopes three physical models made of typical particle size were cast. As depicted in Fig 4, the pure silty soil, fine sand, as well as the mixture sand made of the silty soil and fine sand (1:1 in mass) were adopted. In order to cast these physical models, both the soil blocks and sand collected onsite were air-dried in a shaded room until the moisture content of which does not change. These dried soil blocks were then crushed and separated through a 2-mm-sieve to remove coarse particles. The particle size distribution curves of the samples are presented in Fig 4, Different styles of curves represent the gradation of different soil samples, The small images on the right side of the picture clearly show soil samples of different gradations. These sieved soil masses were adopted to construct the physical models with pure silty soil (H1), horizontal interlayered models made of the silty soil and fine sand composition (H2), and the mixture model made of the silty soil and fine sand composition (H3).

### 2.3. Experimental design

**Fig 5:**
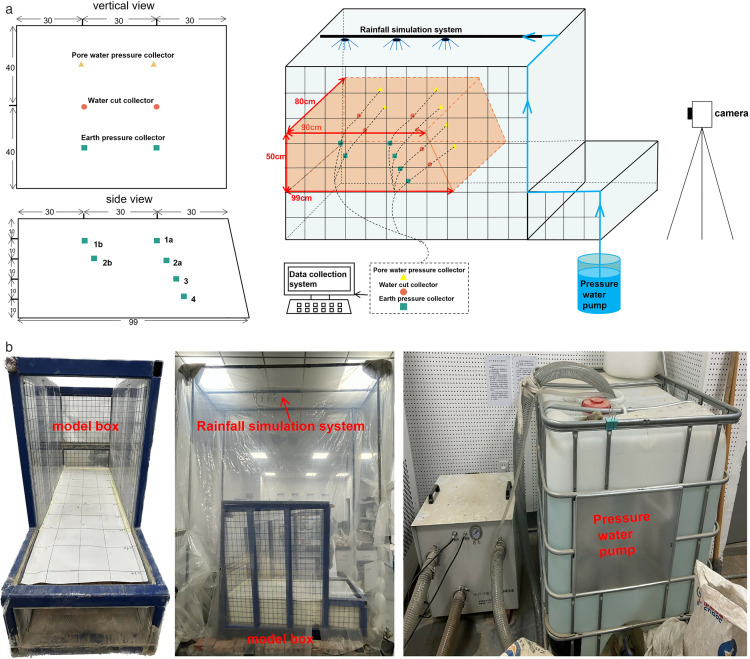
The self-designed instrument. (a) Diagrammatic sketch sensor laying locations. (b) Practicality picture.

The equipment used in the gully erosion tests consisted of a model box, a data acquisition system and an independently designed rainfall simulation device. The rainfall simulation equipment consists of nozzles, water pipes, pressure water pumps and controllers connected together (Fig 5). The rainfall control system adopts NLJY-10 type instrument, which is equipped with three kinds of spray nozzles: 1.5 mm, 3.2 mm and 5.0 mm, the diameter of rain drops from spray nozzles ranges from 0.3 to 6. The spray nozzles are evenly distributed 3m above the soil model to ensure that there is no blind spot of the rainfall area and to meet the requirement of the test, and the spray nozzles are connected to the pump through the water pipe, and the power source of the rainfall system is the water pump, which is connected to the pump through the water pipe. The water pump is the power source of the rainfall system. Using the buttons on the instrument’s computer processing panel, the calculated rainfall intensity in the study area is entered directly into the instrument’s control panel, the water can be discharged by starting the pressurised water pump, and different rain patterns such as light, medium and heavy rain can be simulated and monitored by adjusting the rainfall intensity. The model box used in the experiment measures 2.5 meters in length, 0.8 meters in width, and 5 meters in height. Its base is horizontal, and the sidewalls are constructed of 1 cm thick transparent glass. The exterior side of the glass is embedded with a 0.65 cm x 0.65 cm grid mesh. Raindrops infiltrate the soil model from the top, converging into runoff that drains out from the front of the model box, thus facilitating a clear observation of soil permeation and erosion processes.

The data acquisition system consists of a high precision static strain tester (DH3818) and a water content data collector (CR1000×). The water content sensor (range: 0–100%; accuracy: 0–50%: ± 3%; 50%−100%: ± 5%), pore water pressure sensor (range: −0.01–5Mpa; accuracy: ≤ 0.1%F·S) and soil pressure sensor (range: 0.01–20Mpa; accuracy: ≤ 1%F·S) are connected to the data collector and buried in the soil body. The data collection system is connected to the computer via the data cable and the appropriate software is started to collect the water content value, pore water pressure value and earth pressure value inside the soil body to monitor rainfall infiltration. A row of sensors adjacent to the slope is laid out in four layers on the soil, with a spacing of 10 cm between layers. The outermost row of sensors is positioned 30 cm horizontally from the slope. Due to their greater distance from the slope, the inner row of sensors is only laid out in the top two layers, also with a spacing of 10 cm between layers. The horizontal spacing between the two rows of sensors is 30 cm. Each layer of every row is evenly equipped with three sensors for water content, pore water pressure, and soil pressure. The sensor lines are orderly connected from the right side. The sensor layout diagram is shown in Fig 5. The first layer of sensors is labeled as 1a and 1b, with 1a located on the side closer to the slope. The second layer of sensors is labeled as 2a and 2b, with 2a also located on the side closer to the slope. The third layer of sensors is labeled as 3, and the fourth layer is labeled as 4. Fig 5(a) shows the sensor layout in space and on a plane.In the indoor rainfall simulation tests of the slope model, each test was monitored continuously for 12 days, with water content, pore water pressure and horizontal earth pressure data collected once per second by the monitoring instrument, which was a huge amount of data. Although the monitoring system was stable, there were occasional errors in the large amount of data collected. Because of the continuity of the data, anomalous data can be corrected using data from neighbouring periods. Due to the large data base, the small amount of error after correction has a negligible effect on the overall situation. For statistical analysis and charting, Origin software’s noise reduction feature is used to accurately remove individual anomalies, ensuring accurate and reliable data charts and providing a solid foundation for research and analysis.

Based on previous studies, under the same rainfall amount and slope morphology, due to the limited height of artificial rainfall and the higher concentration of raindrops, the soil erosion amount in artificial rainfall simulation tests is less than half of that under natural conditions. According to the data recorded by rainfall sensors at sampling points in the study area, the maximum daily rainfall is 69.1 mm. Using the similarity ratio, the daily artificial rainfall is calculated to be 1.38 mm, with a rainfall intensity of 10 mm/h. To ensure that the rainfall erosivity in the model test is comparable to that of natural rainfall, the model’s rainfall amount is designed to be doubled, resulting in a daily rainfall of 2.76 mm. The daily rainfall begins at 10 am and lasts for 17 minutes. The experiment is conducted for a maximum duration of 12 days, with a cumulative rainfall of approximately 563 mm. During the rainfall process, erosion characteristics are recorded, and the water content, pore water pressure, and soil pressure are monitored in real-time using the data acquisition system.

### 2.4. Experimental procedure

**Fig 6:**
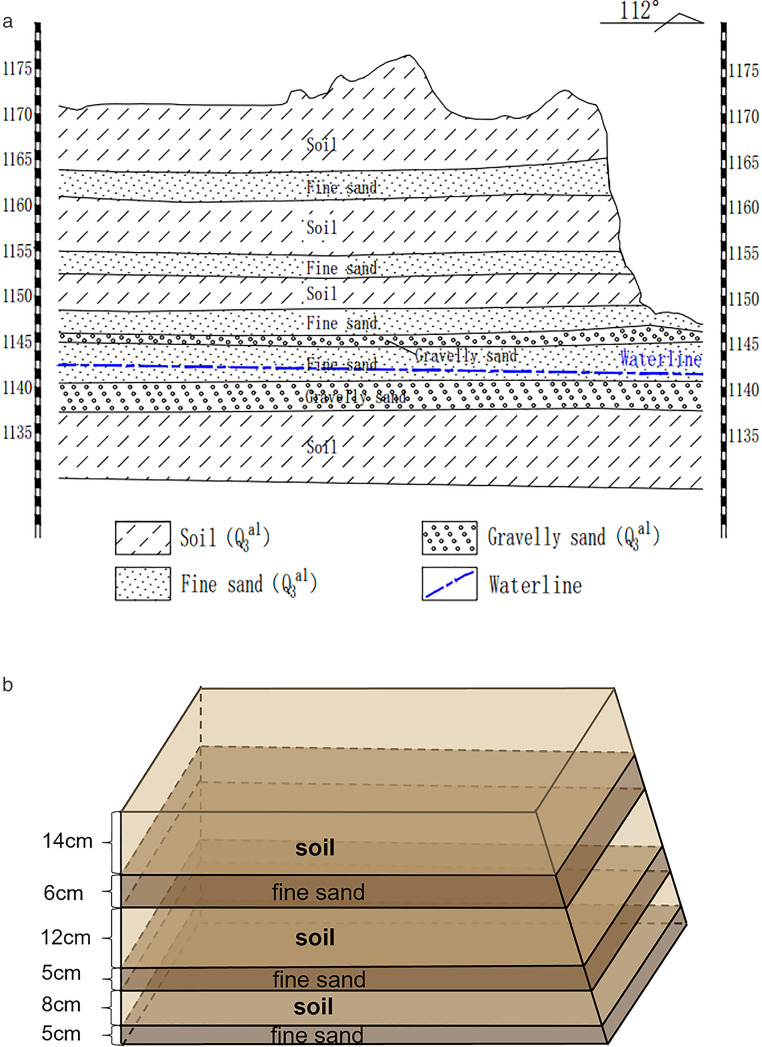
Profile of sampling points and generalised thickness of soil layers. (a) Sampling point profile. (b) Thickness of different layers.

Three sets of soil slope model tests with varying particle size compositions were conducted: a pure silt soil slope model (H1), a horizontally interbedded silt and fine sand soil slope model (H2), and an equally mixed silt and fine sand soil slope model (H3). Based on the actual stratigraphic profile (Fig 6(a)), the thickness of each actual soil layer was delineated and then the height of the model was generalised according to a 1:50 similarity ratio. Create a horizontal inter-layered soil slope model (H2) composed of powdered soil and fine sand, generalise the layering thickness of each powdered soil and fine sand layer of the indoor model according to the actual thickness of each layer, and control the density of the soil layer by beating the soil layer according to the density of the in-situ soil as in Fig 6(b). The slope angle of the models was approximately 80 degrees. The “Kapo” top platform exhibits a wide distribution, with slope lengths reaching several kilometers, and similar gully erosion mechanisms observed on the slopes. The soil slope models were constructed based on the actual conditions of the study area and were studied from a two-dimensional perspective. Based on the actual soil slope, with a sampling point slope height of 25m, the dimensions of the experimental soil slope model were designed to be 1.2m long, 0.5m high, and 0.8m wide, with a similarity ratio of 50. The slope was designed by scaling down the corresponding proportions according to the similarity ratio [31]. The natural soil moisture content was 9%. Comparison table of test parameters (Table 4).

**Table 4:** Comparison table of test parameters.

Categories	Particulars
Slope soil types	Pure chalk slope, chalk and fine sand horizontal interlayer slope, chalk and fine sand equal ratio uniform mix slope
Intensity of rainfall	10 mm/h
Daily rainfall	2.76 mm
Duration of the rainfall	Rainfall of 17 min per day for 12 days, with a cumulative rainfall of about 563 mm.
Particle size distribution	Powdery soil and fine sand were sieved through a 2 mm sieve to remove coarse particles and used for the test; powdery soil samples, fine sand samples, and powdery soil and fine sand mixed soil samples in equal proportions each had their own particle size distribution curves (Fig 5).
Monitoring data	Water content, pore water pressure, horizontal earth pressure
Frequency of data monitoring	Reads a value every second
Modelled slope gradient	Nearly 80 °
Model size	Length 1.2m, height 0.5m, width 0.8m
Water content of in-situ soils	9%

To minimize the differences in properties between the experimental soil and natural soil and ensure that the moisture content and density of the soil slope model matched those on-site, the soil was first air-dried and mixed to achieve a 9% moisture content prior to constructing the model. The soil samples were then covered with plastic wrap and allowed to rest for one day to ensure a uniform moisture content. The density of each soil layer model was controlled by the compaction level of each soil layer during the construction process, ensuring a consistent ratio of mass to volume. To mitigate the influence of boundary constraints on the soil model, Vaseline was applied to the inner walls of the model box to reduce friction between the sidewalls and the experimental soil. Subsequently, a layer of fine sand was placed on the bottom of the model box to allow for even water penetration into the soil. To ensure a relatively uniform and continuous soil structure in the model, each 5 cm layer of soil was compacted before adding the next layer, and the surface was lightly scarified before continuing. After completing the construction of the soil slope model, it was left to stand for 2 days prior to conducting the experiment. During this period, the model was covered with plastic film to reduce water evaporation.

## 3. Results

### 3.1. The evolution of slope erosion

Under the same rainfall intensity, the erosion and evolution of the three sets of soil slopes were analyzed, as shown in Table 5. The process of gully erosion and destruction follows the sequence of local damage, toe damage, mid-slope damage, upper slope damage, and finally crest damage. Raindrop splash erosion, sheet erosion, and rill erosion are the main causes of slope erosion and destruction. All three models began to exhibit local damage on the fifth day of the test and entered an accelerated damage phase on the seventh day. Initially, raindrops impact the slope surface, displacing surface soil particles without causing any noticeable deformation of the slope. Subsequently, sheet erosion removes loose particles on the slope surface, leaving shallow erosion marks. Local damage then occurs on the slope. Finally, under the effect of rill erosion, gullies form on the slope, resulting in erosion and destruction progressing from the toe to the crest of the slope in a retrogressive manner. Soil with a higher content of coarse sand particles is more resistant to raindrop impact, resulting in shallower gully erosion depths. In contrast, soil with a higher content of silt is more prone to being carried away by rainwater, leading to more severe gully erosion.

**Table 5:** 3-group physical modeling of rainfall erosion damage processes.

**H1**	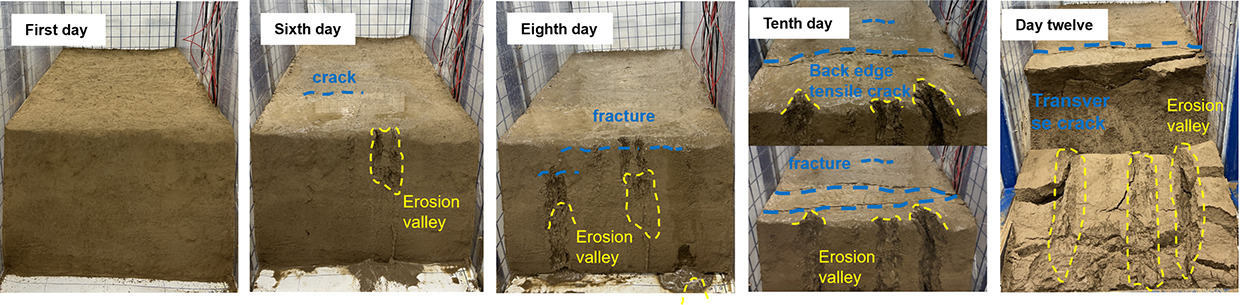
**H2**	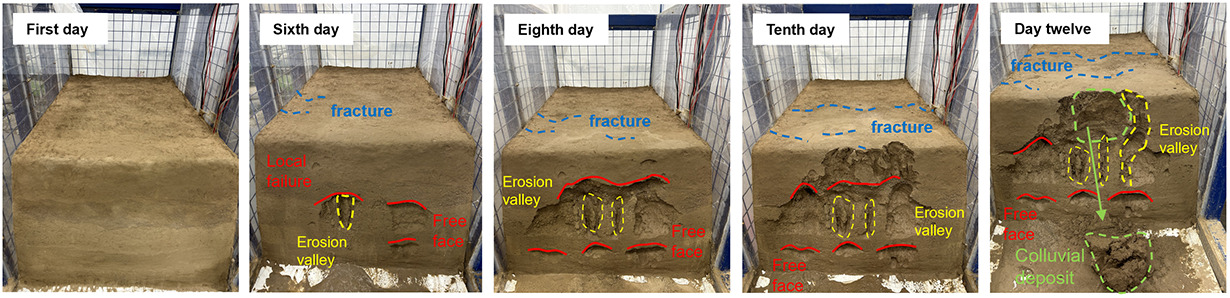
**H3**	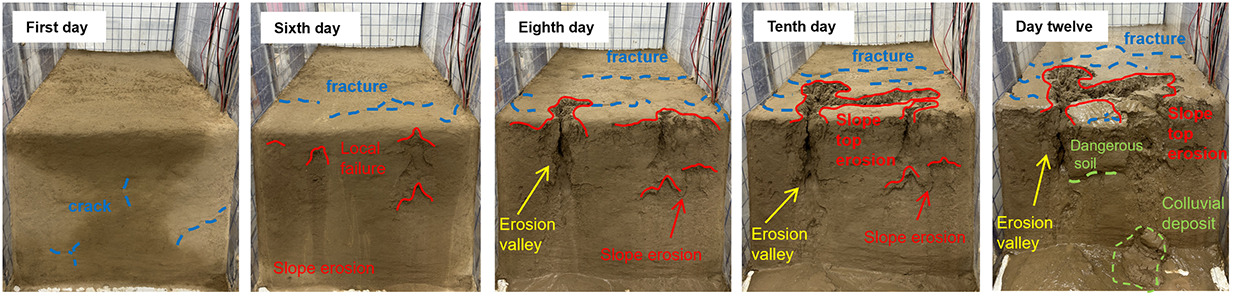

The H1 model experienced the most severe damage. Due to the low permeability of silt, most of the rainwater quickly flowed towards the slope toe, increasing the water content and reducing cohesion, thereby leading to erosion and damage. The erosion gully extended from the slope toe up to the slope crest. The silt slope base was weak, and the slope toe was prone to spreading. Slope failure often involved the base, and under the action of its own gravity, a trailing tension crack formed at the slope crest, penetrating the soil and resulting in a landslide.

The initial local damage occurred in the H2 model, specifically in the soil layer beneath the sand layer, and gradually expanded due to water erosion. Given that the permeability of fine sand is greater than that of silt, rainwater infiltrates from the soil layer into the sand layer, where it is absorbed, increasing the water content without reaching saturation. Water forms a thin film around the sand particles, enhancing their cohesion. As rainwater infiltrates from the sand layer back into the soil layer, the soil’s permeability decreases, preventing all rainwater from infiltrating rapidly. Consequently, rainwater accumulates at the sand-soil interface, increasing the water content of the soil particles there. This, in turn, thickens the water film around soil particles, increases the distance between them, reduces cohesion and the internal friction angle, and decreases the soil’s resistance to shear failure, ultimately leading to erosion and damage. The damage to the soil layer beneath the sand layer gradually increases, expanding and deepening inward and forming a free face on the slope. When rainwater reaches this free face, it erodes into the soil mass along the face, washing away soil particles and a small amount of sand particles at the sand-soil interface. The free face gradually enlarges, reducing the surrounding support. The water content of the soil mass above the free face gradually increases, increasing its weight, and tension cracks appear above it. These cracks expand and connect with the lower free face, providing an effective channel for rainwater drainage. The soil structure is destroyed, leading to erosional collapses under the action of its own gravity.

The H3 model contains fine sand with large pores between particles, making it susceptible to drying and surface cracking. The silt particles on the slope crest are easily carried away by sheet and rill flows, exposing the coarse sand particles. The destruction of the structure between the coarse sand particles and the surrounding soil leads to their erosion and transport by water flow, resulting in severe erosion and damage at the slope crest. As the rainwater carries the eroded sand and soil, its hydrodynamic force diminishes. The coarse sand particles on the slope effectively block the impact of the sand and soil carried by the rainwater, resulting in reduced erosion on the side slopes.

Soil permeability determines how quickly and how much rainfall infiltrates into the soil. Good permeability allows rainwater to infiltrate quickly into the ground, reducing surface runoff. Fine particles, small pores and poor connectivity, low permeability of the soil, rainfall, most of the rainwater can not penetrate the soil in a timely manner, the formation of stagnant water on the surface, will reduce the cohesion between soil particles to reduce the soil structure has become loose, stagnant water in the formation of runoff under the action of gravity, runoff has a certain amount of energy, it will be scoured the soil surface, the loose soil particles are more likely to be taken away, so that erosion is aggravated. The type of erosion also varies according to the permeability of the soil. In areas of low permeability, sheet and gully erosion are the main types. Sheet erosion is a phenomenon in which thin layers of soil on the surface are uniformly washed away and lost by runoff from the slope, which can easily result in large areas of soil loss due to the wide distribution of runoff on the surface and the low resistance of the soil to scouring. Gully erosion, on the other hand, is a form of erosion where runoff is concentrated on the slope surface and the energy of the water flow is high, scouring the soil into a gully. In contrast, in areas with more permeable soils, soil erosion is relatively mild because most of the rainwater has infiltrated into the ground, reducing the scouring power of surface runoff.

The presence of coarse sand increases the porosity within the soil, providing more space for rainwater to infiltrate. In a sandy soil layer, the larger particle size of the sand and good pore connectivity allow rainwater to infiltrate quickly into the pore space, reducing the generation of surface runoff. This reduces the scouring energy of the runoff on the soil surface and reduces erosion. Coarse sand on the soil surface can effectively resist the impact of raindrops. Raindrops have a certain amount of kinetic energy when they fall, and when they impact directly on fine-grained soils, they destroy the soil surface structure, causing soil particles to disperse and be easily carried away by runoff. While coarse particles form a kind of ‘protective layer’ on the soil surface, some of the energy is dissipated and absorbed when raindrops strike coarse particles, reducing the damage to the soil caused by initial rain erosion and reducing the damage to the underlying fine-grained soil. Coarse and fine particles mix and the coarse particles are relatively stable and can limit the movement of the fine particles. When runoff occurs, the fine particles are driven by the water flow, while the coarse particles can play a blocking and fixing role, preventing the fine particles from being washed away in large quantities, thus slowing the rate of soil erosion.

### 3.2. Morphological characteristics of gully erosion

**Fig 7:**
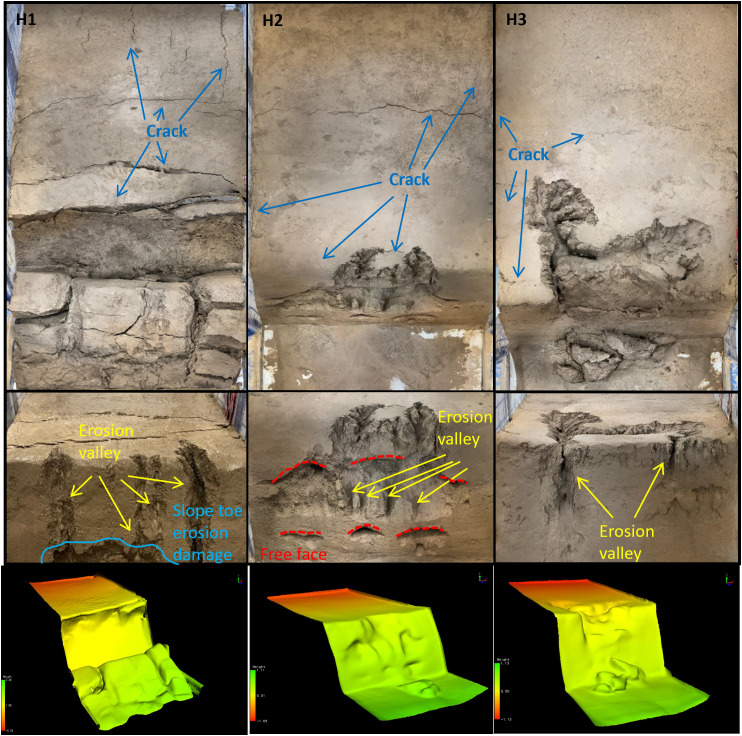
Overall morphological characteristics of gully erosion.

The degree of erosion on the surfaces of the three experimental slopes under rainfall was analyzed using a combination of qualitative and quantitative methods. Using the panoramic views and 3D laser scanning images in Fig 7, the morphology of slope erosion and gully damage can be clearly observed.

Fine particles have a large specific surface area and high surface activity. As rainfall increases and the water content of the soil changes, the contraction and expansion of fine clay particles become more significant. During the dormancy process, the clay particles lose water and shrink, creating a large internal stress due to the mutual constraints between the particles. This stress is concentrated in certain weak areas, and when it exceeds the tensile strength of the soil, it triggers the formation of cracks. Soils with high levels of fine particles have poor permeability and slow water movement. After rainfall or irrigation, the water content of the surface soil increases rapidly, while the deep soil moisture changes relatively slowly, resulting in a large difference in water content between the upper and lower layers of the soil, causing uneven expansion and deformation, and further promoting the formation and expansion of cracks. Coarse grained sands and soils are equivalent to skeletal structures in the soil. On the one hand, their presence can increase the porosity of the soil so that the soil has more space to accommodate the inward and outward movement of water during the dry-wet cycle, buffering the volume change of the soil to some extent and reducing the stress concentration caused by the water change, thus inhibiting the formation of cracks. On the other hand, once cracks have formed, coarse particles can inhibit further crack propagation. Due to the large particle size of coarse particles, when the crack encounters coarse particles in the expansion process, it has to bypass them and change the direction of expansion, which consumes the energy of crack expansion and reduces the expansion speed, and the length and width of the crack are relatively small, and the degree of expansion is limited. When coarse and fine particles are mixed in the soil, stress concentration points are easily formed at the junction of particles of different grain sizes. Due to the different expansion and contraction characteristics of powdery and sandy soils, a greater stress will be generated at the interface between the two as the moisture changes, thus initiating cracking. In summary, the degree of cracking H1 is greater than H2 is greater than H3.

H1 suffered severe damage at the slope toe, with long cracks developing throughout, leading to the first collapse. The runoff erosion gullies on H1’s slope were the deepest, extending from the slope toe to the top, with landslides occurring at the long cracks situated approximately 1/5 of the way from the outer edge of the slope. H2 experienced the first slope surface erosion and damage, with the largest erosion area in the upper-middle section of the slope, accounting for approximately 2/3 of the total slope area. Due to the erosion of the middle soil layer, four overhanging sections were formed on H2’s slope. The erosion gullies on H3’s slope top were the deepest, with the largest erosion area covering approximately 1/3 of the entire top surface. The erosion depth accounted for half of the slope height.

At the end of the experiment, on the slope crest, the number of long cracks decreased with the increase in coarse sand content, while the number of microcracks increased. Specifically, H1 had 4 long cracks, H2 had 2 long cracks, and H3 had 1 long crack and over 10 microcracks. Vertically, as the coarse sand content increased, the length and depth of gully erosion decreased, and the number of rills formed by erosion also decreased. H1 had 4 long gullies, H2 had 2 short gullies, and H3 had 2 short gullies. Horizontally, the erosion pattern of the gullies developed toward the rear of the slope, resembling the growth of tree roots. With the increase in coarse sand content, the erosion damage expanded further toward the rear. The damage length on the slope crest was approximately 5 cm for H1, 10 cm for H2, and 30 cm for H3. The erosion and damage of pure silt slopes are the most severe.

### 3.3. Rainfall-induced erosion modifies soil properties

**Fig 8:**
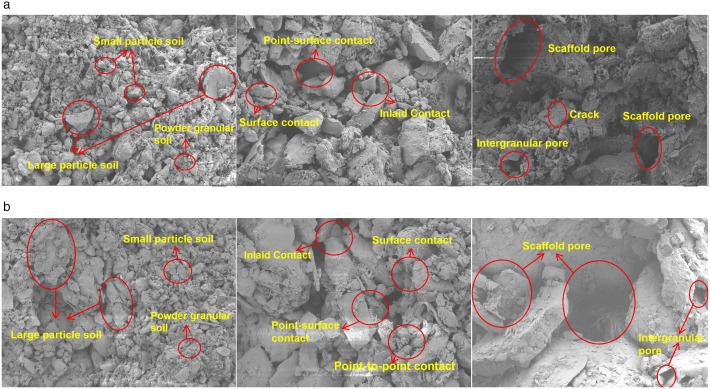
Microscopic pore changes in soil mass. (a) Intergranular pores of undisturbed soil. (b) Intergranular pores at saturated water content.

Under rainfall conditions, the soil moisture content of the slope increases, and both soil density and strength exhibit an initial rise followed by a decline. At low moisture contents, a thin water film forms between soil particles, acting as a binder and increasing the cohesion and internal friction angles of the soil mass. Consequently, soil strength improves with increased compactness. However, at high moisture contents, the water film increases the distances between particles, reducing soil cohesion and internal friction angles. Once the soil compactness reaches a certain level, it begins to decrease, and soil strength correspondingly diminishes with the reduction in compactness. The shear strength of the soil mass decreases with increasing moisture content, rendering it susceptible to erosion and damage. Fig 8(a)–8(b) illustrate the changes in soil microstructure morphology at different moisture contents. Under rainfall conditions, variations in soil moisture content and pore water pressure lead to changes in particle positions and rearrangements of the contact relationships between particles. Some silt particles form aggregates, whereas some fine-grained sand and soil are carried away by rainwater through large pores or cracks. Consequently, the number and size of pores between particles increase significantly, enhancing the connectivity among pores and reducing the contact relationships between particles. This leads to decreased shear strength and reduced stability of the soil mass.

### 3.4. Response variation parameters to rainfall

Origin software has powerful data analysis and visualization capabilities, enabling efficient and accurate curve fitting and statistical analysis. By using Origin software to process a large amount of experimental data from monitoring, it was found that rainfall intensity and duration are closely related to critical rainfall intensity. Rainfall intensity and duration are closely related to critical rainfall intensity. High-intensity, short-duration rainfall can saturate slopes in a short period of time, triggering collapse and erosion; low-intensity, long-duration rainfall that leaves the soil waterlogged for a long period of time will also gradually reduce the shear strength of the soil, ultimately leading to slope destabilisation. In areas of high rainfall frequency, the rock and soil bodies of slopes undergo frequent wet and dry cycles and their physical and mechanical properties deteriorate, and the critical rainfall intensity decreases accordingly. Under rainfall conditions, as rainwater infiltrates the soil body, the increase in soil moisture content leads to a rise in pore water pressure, which in turn increases the internal soil pressure. When the soil reaches its saturation point, the pore water pressure exceeds the soil’s bearing capacity, causing a decrease in soil strength, which subsequently leads to a reduction in internal soil pressure and a decrease in slope stability, ultimately triggering landslide events. By integrating the changes in moisture content, pore water pressure, and internal soil pressure, we can analyze the alterations in the internal soil structure during the process of external erosion and damage.

#### 3.4.1. Changes of moisture content.

As the moisture content increases, the internal friction angle and cohesion initially rise and then decline. When the moisture content is low, a thin water film forms between particles, acting as a bonding agent that enhances the soil’s cohesion and internal friction angle. This occurs prior to rainfall affecting the slope, resulting in a dense surface layer. As the moisture content increases, the bonding water film between soil particles thickens, acting as a lubricant, thereby reducing the friction, increasing the distance between particles, and decreasing the cohesion among them. Subsequently, soil particles are carried away by rainwater, leading to the erosion and damage of the soil body.

**Fig 9:**
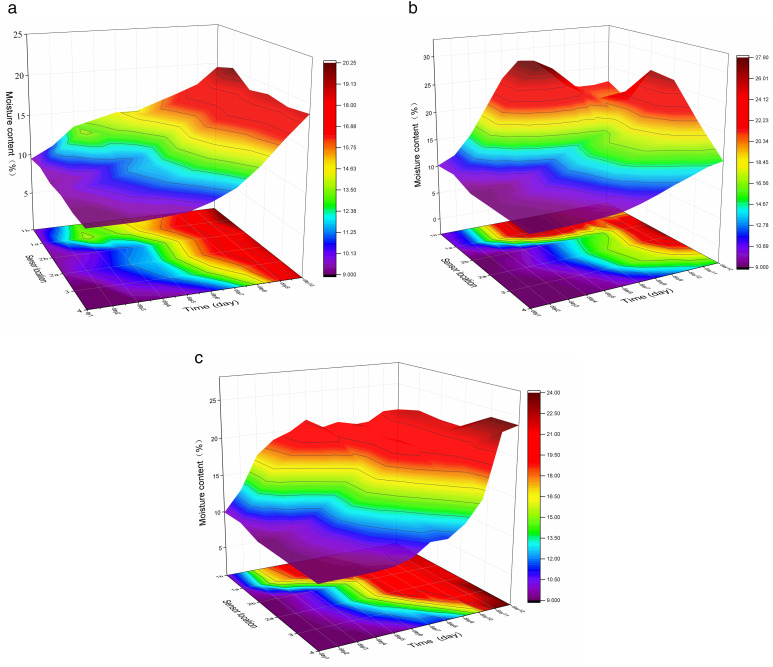
Contour map of soil slope moisture content variation. (a) Model-H1. (b) Model-H2. (c) Model-H3.

As illustrated in Fig 9, within a single-day rainfall cycle, when rainfall infiltration commences, the wetting front reaches the sensor, and the curve typically exhibits a rapid increase in water content. Subsequently, as the rainwater continues to infiltrate, the water content decreases and eventually stabilizes, awaiting the subsequent rainfall infiltration cycle. The outer sensor detects changes in water content sooner than the inner sensor, indicating that the outer soil receives rainwater infiltration from both vertical and lateral directions. The permeability of H1 is uniform, and the water content initially rises until it approaches saturation. When the rainfall accumulates to 282 mm and the water content of the upper soil layer reaches 14.7%, erosion and damage commence on the slope surface, becoming more severe as the water content increases. The first-layer sensor of H2 is situated in the sand layer, where permeability and porosity are higher than those of silt. In the initial stage, water infiltrates slowly from the sand layer into the underlying soil layer, causing a rapid increase in the sand layer’s water content and a gradual increase in the water content of the lower layer. When the rainfall accumulates to 235 mm and the water content of the upper soil layer reaches 27.9%, local damage initiates on the slope. In the later stages, due to the formation of cracks within the soil, the infiltration of rainwater accelerates, leading to a decrease in the water content of the upper sand layer and an increase in the water content of the lower soil layer. The higher the water content in the sand layer, the more severe the damage to the underlying soil layer. The permeability of H3 is uniform, and the water content of each layer of sensors first increases and then stabilizes. When the rainfall reaches 258 mm and the water content of the first layer stabilizes at 21.6%, local erosion and damage occur on the slope surface. Micro-cracks appear on the top surface of the slope, providing preferential pathways for rainfall infiltration. The water content of the lower soil layer continues to increase, resulting in severe erosion and damage to the slope. Therefore, the erosion and damage mode of the slope varies with the distribution of water content and preferential infiltration paths.

#### 3.4.2. Changes of pore water pressure.

**Fig 10:**
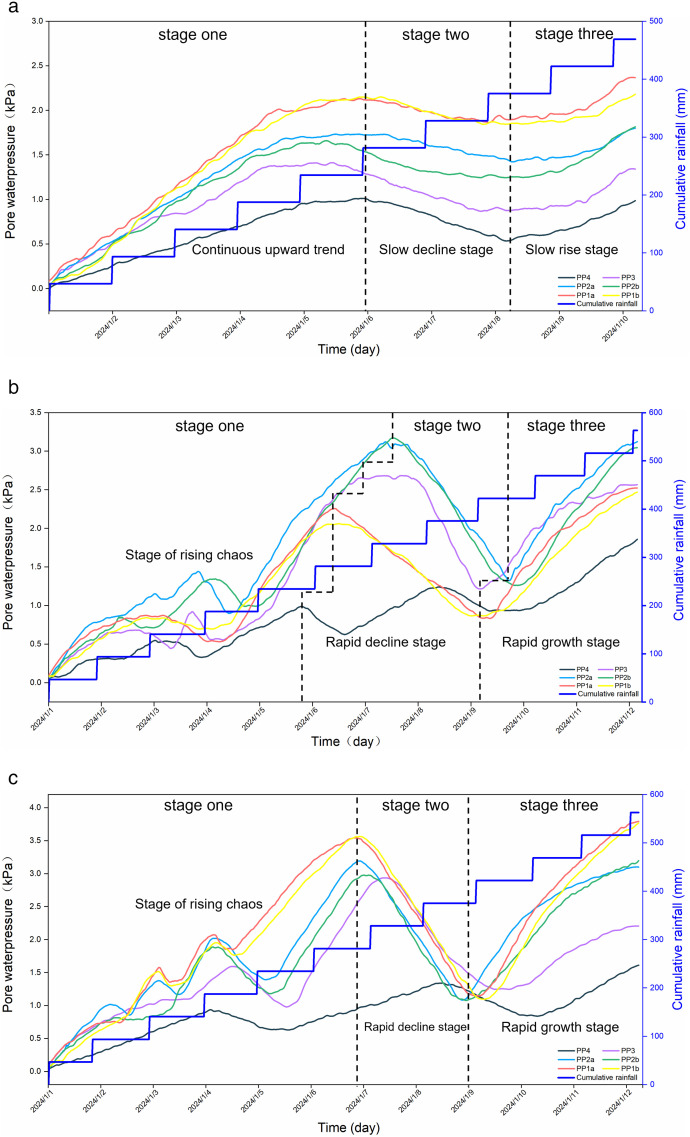
Variation of pore water pressure of soil slope with rainfall profile. (a) Model-H1. (b) Model-H2. (c) Model-H3.

As depicted in Fig 10, the pore water pressure at the outer sensors responds most rapidly, as rainwater initially infiltrates the outer soil body, increasing the moisture content and consequently elevating the pore water pressure. Throughout the rainfall erosion experiment, the changes in pore water pressure can be divided into three stages: Firstly, as rainwater gradually migrates towards the detection points, the soil moisture content gradually increases, causing fluctuations and a rise in pore water pressure. Secondly, as soil erosion and damage progress, internal cracks develop, permitting water within the soil to transport fine-grained soil particles downward through these cracks. This leads to a slight increase in soil moisture content and the expansion of micro-pores within the soil, resulting in a decrease in pore water pressure. However, due to the slow migration of water in fine-grained soils, the decline in pore water pressure is gradual. Thirdly, as cracks propagate, the soil moisture content gradually reaches saturation, leading to an increase in soil pore water and subsequently pore water pressure, which gradually rises until the experiment is halted.

As shown in Fig 10(a) and (c), the soil permeability of H1 and H3 is uniform, allowing rainwater to infiltrate gradually. Consequently, the moisture content in the upper layers of the soil body is higher than in the lower layers, leading to greater pore water pressure in the upper layers compared to the lower layers. In contrast, Fig 10(b) indicates that for H2, due to the excellent water permeability and high infiltration rate of the sand layer, rainwater infiltrates rapidly, leading to an increase in moisture content and subsequently in pore water pressure. The silt layer, however, has poorer permeability than the sand layer, resulting in a slower increase in pore water pressure. Due to the formation of microcracks within the soil, erosion occurs, causing fluctuations in the pore water pressure curve. Although this study revealed the dynamic response patterns of pore water pressure through multi-stage experiments, the model has inherent limitations in processing time series data. Newly added monitoring data may be difficult to integrate uniformly and standardize using the existing model, potentially leading to fragmentation of the dynamic database and affecting the in-depth analysis and pattern transfer of long-term monitoring data.

#### 3.4.3. Changes of soil pressure.

As rainfall begins to infiltrate, gravitational stress is imparted to the soil, and the internal horizontal earth pressure sensors respond more rapidly than the moisture content and pore water pressure sensors. The overall trend of the earth pressure curve initially rises and then levels off. Prior to slope erosion and failure: As rainfall infiltrates, the soil moisture content and pore water pressure increase, leading to a gradual rise in earth pressure. Partial erosion and failure of the slope: Internal cracking leads to a smaller increase in moisture content and an increase in internal soil pores, which in turn reduces pore water pressure. The earth pressure in the upper layer fluctuates and rises; as rainwater continues to infiltrate, the earth pressure in the lower layers becomes greater than that in the upper layer. Severe erosion and failure of the slope: The internal soil moisture content gradually reaches saturation, leading to a continuous increase in pore water pressure. The earth pressure rises slowly and then levels off. Erosion on the outer side of the soil is severe, with cracks developing that disrupt the soil structure. This results in lower earth pressure on the outer side of each layer compared to the inner side, while the earth pressure on the inner sensors increases gradually.

**Fig 11:**
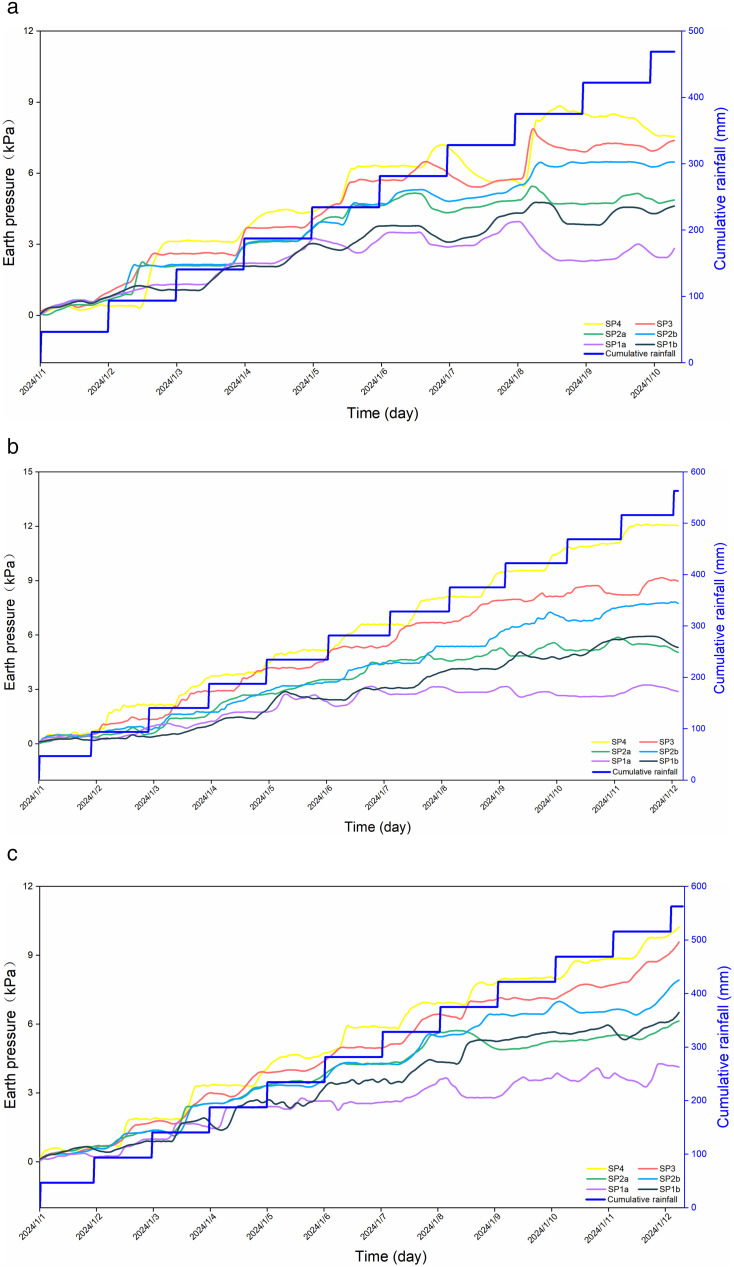
The relationship between the soil pressure and rainfall duration. (a) Model-H1. (b) Model-H2. (c) Model-H3.

As shown in Fig 11(a), for H1, due to the development of through-cracks in the later stages, slope failure progresses from the toe to the crest, resulting in a downward trend in earth pressure. Fig 11(b) and (c) indicate that for H2 and H3, slope failure initiates from the middle of the slope and progresses toward the crest, with the earth pressure at the lower part of the slope continuing to increase.

As seen in Fig 11(a), for H1, due to the development of through-cracks in the later stages, slope failure occurs from the toe to the crest, resulting in a downward trend in earth pressure. Fig 11(b) and (c) show that for H2 and H3, slope failure initiates from the middle of the slope and progresses towards the crest, with the earth pressure at the lower part of the slope continuing to increase. Analysis indicates that when slope failure occurs, the soil moisture content nearly reaches saturation, and soil deformation and compression result in an increase in internal pore water pressure. The increase and concentration of horizontal stress cause significant changes in the corresponding earth pressure, suggesting that horizontal earth pressure plays a crucial role during slope failure.

## 4. Discussion

Based on the indoor rainfall simulation test, three groups of control tests were designed for the stratigraphic sequence of the soil in Wensu County: no coarse-grained slopes, coarse-fine-grained interbedded slopes, and coarse-fine-grained isoparametric homogeneous mixed slopes. The effects of soil type, grain size, pore structure and water content on the critical rainfall intensity were investigated. Sandy soils have a high permeability and rainfall is easily infiltrated, requiring a high intensity of rainfall for a short period of time before collapse and erosion can be triggered; whereas clay soils have a low permeability and erosion can be triggered by rainfall converging on the surface to form runoff with a low intensity of rainfall. If the ratio of coarse to fine particles is appropriate, a relatively stable structure can be formed to resist a certain amount of rainfall erosion; if there are too many fine particles, the soil structure can be destroyed at lower rainfall intensities. The study did not carefully quantify the effect of coarse particle content on slope erosion rates. Follow-up experiments should be designed for slopes with different coarse particle contents to investigate their rainfall erosion resistance characteristics and to determine the optimum content ratio.

Based on the results of research into the effects of soils with different grain size compositions, cracks and internal pressure on slope erosion, appropriate protective measures are provided for the excavation and protection of slopes in engineering works. For example, in the area with high coarse sand content, given its high resistance to erosion, coarse sand bedding can be used to improve the ability of the slope to resist the impact of raindrops and slope runoff. On the other hand, in areas with high chalk content and poor stability, methods such as shotcrete berms are used to prevent wash-out erosion. To reduce the scouring of the slope by slope runoff, a water interceptor ditch is constructed at the top of the slope to intercept incoming water from above the slope to prevent prolonged accumulation of water on the slope.

### 4.1. Effects of different grain size soils on slope erosion

The test shows that the erosion damage of rainwater on the slope can be divided into three stages: raindrop impact, surface flow erosion and runoff erosion. Different particle size composition of the soil body slope resistance to erosion is different, coarse sand content increases, slope resistance to raindrop impact and slope runoff ability to increase the erosion depth is small, the destruction of a long time. Fine sand particles large, high porosity, high internal friction angle, conducive to resisting water flow; chalk soil containing colloidal material, swelling when wet, reducing soil stability, chalk soil slope gully erosion is significant. Through the experiment, the specific response mechanism of soils with different grain sizes in the process of rainwater erosion of slopes was analysed in detail, and the development of slope erosion was elaborated in a comprehensive way, from the impact of raindrops, water infiltration to the formation of slope runoff, and so on. In recent years, researchers have focused on the changing patterns of infiltration and runoff coefficients of different soil types with rainfall intensity and slope, as well as the determination of critical slopes through modelling experiments [32–34]. However, this paper focuses on the erosion performance of soils with different grain size composition, especially coarse-grained and fine-grained interbedded soil slopes, under the effect of rainfall intensity and runoff, and the entry point focuses more on the effect of differences in soil internal structure on erosion. This study can help to understand the internal mechanism of slope erosion and provide a theoretical basis for the protection of slopes with different grain size compositions.

### 4.2. Effect of crack development on slope erosion

The experimental study found that geological and structural factors, such as a high degree of weathering and many cracks in the slope, cause the soil to absorb water and soften and reduce the shear strength during rainfall, with a consequent reduction in the critical rainfall strength. During the rainfall process, the slope surface is saturated due to the difference in water content distribution, resulting in micro-cracks at the top of the slope and the slope surface, which accelerate the erosion and damage of the slope, and they provide channels for rainwater infiltration, triggering the latent erosion, resulting in the widening and deepening of the cracks, changing the hydrological characteristics and strength of the soil, and ultimately causing the slope to erode and damage the slope from the foot of the slope to the top of the slope. Through an in-depth and systematic study of the dynamic mechanism of cracks in the process of slope erosion and damage, the causes of cracks, the effects of water infiltration and the effects of slope stability damage are explained in detail. Previous authors have focused on the control of shallow and deep penetrating cracks on platform edges by rainfall, the failure modes of cracks leading to cracks in different contexts, the size of landslides triggered and the dual effect on slope stability [35–37]. This experiment is based on the example of Kabu in Wensu County, focusing on the study of the whole process of slope erosion damage by geological structures and cracks under heavy rainfall conditions, which is a broader research object and focuses on the dynamic process of erosion damage rather than the failure of cracks in specific areas, laying the foundation for the subsequent study of slope stability problems under complex geological conditions.

### 4.3. Effect of internal soil pressure on slope erosion

Changes in the grain size composition and internal structure of soil slopes under rainfall conditions significantly affect the extent of slope erosion damage. In stratified sedimentary soil slopes, rainfall induced seepage erosion accelerates water infiltration, and seepage induced internal erosion affects the slope damage pattern and mechanism, and such slopes may fail gradually and the damage is difficult to predict after repeated rainfall. This experiment systematically studies the influence of multiple factors on soil erosion, not only from the water content, pore water pressure and horizontal soil pressure and other internal pressure factors in-depth analysis, but also combined with rainfall on the role of layered sedimentary soil slopes to carry out the study, the establishment of simulations to verify the research methodology is scientifically rigorous, and the conclusions have a strong persuasive power. However, previous studies have addressed the effect of rainfall on slope erosion by constructing a coupled seepage-erosion finite element model and verifying it with simulations [38,39]. On this basis, this paper focuses on the specific type of laminated sedimentary soil slope, analyses its internal physical processes and the combined effect of internal pressure factors, and carries out research on the hidden nature of internal erosion, impact and related processes, which are difficult to assess, making the research object and focus more unique compared to other studies.

### 4.4. Time dependent rainfall suffers from uncertainty and seasonality

This study achieved similarity to field conditions by controlling rainfall intensity and slope physical parameters; however, the single time series of artificial rainfall differs fundamentally from the randomness of natural rainfall (e.g., intermittent rainfall).The “intermittent periods” of rainfall significantly affect soil infiltration-evaporation balance, thereby altering the rate of decline in slope shear strength [40]. However, this experiment did not account for such time-dependent rainfall processes, which may limit a comprehensive understanding of the dynamic response mechanisms of slope erosion. Additionally, although the “doubling rainfall” method was used to correct region-specific rainfall data, this method did not account for differences in raindrop kinetic energy across seasons [41]. This may limit the applicability of conclusions such as the erosion threshold for coarse-fine interlayered slopes to specific rainfall types, making them difficult to generalize to seasonal rainfall scenarios throughout the year. The time-dependent and seasonal variability characteristics of natural rainfall, as well as the potential underestimation of the cumulative effects of pore water pressure by short-term monitoring data, combined with instrument errors during data collection, may all lead to inaccuracies in determining the critical rainfall intensity. Therefore, subsequent studies should integrate time-dependent rainfall-seepage-erosion coupled numerical simulations to quantify the impact of rainfall data uncertainty on slope erosion predictions, thereby enhancing the reliability of conclusions.

## 5. Conclusions

Using the case of card-breaking erosion in Wensu County, this study reveals the damage patterns and damage mechanisms of slopes with different grain size compositions under rainfall conditions. It provides guidance for local slope control. The following conclusions are made:

(1) The interface between the sand layer and the soil layer can easily form a critical surface due to the difference in permeability. Coarse-grained sand has strong shear resistance to runoff, and the slope containing coarse-grained sand forms runoff earlier, reducing rainfall infiltration and slowing the formation of erosion gullies; pure soil slopes have rapid increase in water content and loose surface particles, resulting in lower cohesion and internal friction angle, which are susceptible to raindrop splashing and runoff erosion, and rainfall erosion is severe;
(2) When rainwater infiltrates, the water content of the soil body increases, the pore water pressure first rises, then falls and rises again, and the horizontal earth pressure first increases rapidly, then slowly. Cracks for rainwater infiltration to provide an advantageous path, resulting in the surrounding fine-grained soil transported with the water, soil body internal latent erosion, cracks increase, soil structure was damaged. Under the action of both, the internal horizontal stress increases, and the slope becomes unstable;
(3) The mechanism of rainwater erosion on slopes is raindrop impact, slope surface flow erosion and run-off erosion. Slope damage is regressive, in the order of localised slope face, toe of slope, central, upper and top face damage. Slopes with coarser particles are more resistant to erosion;
(4) Chalk slope is very prone to gully erosion disaster, the construction of drainage channels at the top of the slope, to reduce rainwater infiltration into the slope, to avoid latent erosion phenomenon. The junction of pulverised soil and fine sand is easily eroded into the airspace, and it is possible to hang the net and spray slurry on the pulverised soil layer to reduce the loss of pulverised soil and strengthen the stability of the slope.

## Supporting information

S1 File The foundational image data generated by the 3D laser scanning software used to create the supporting data and plotted images for the particle size distribution curves of the four samples. (ZIP)

S2 File The raw data recorded during the experiment for generating Figs 9–11, including pore water pressure sensors, horizontal soil pressure sensors, and moisture content sensors. (ZIP)
