# Correction: Effects of soil particle size on rainfall-induced erosion of near-horizontal layered slopes

**DOI:** 10.1371/journal.pone.0343821

**Published:** 2026-02-25

**Authors:** Chenxin Liu, Zizhao Zhang, Lilong Cheng, Yamei Wang, Xinyu Liu, Runsen Lai, Qianli Lyv

The authors, Chenxin Liu, Lilong Cheng, Yamei Wang, Xinyu Liu, Runsen Lai and Qianli Lyv, should not have been attributed equal contribution to this work.
