# Supplementary figures and images for: Effects of soil particle size on rainfall-induced erosion of near-horizontal layered slopes
Source: PLoS One. 2025 Sep 22;20(9):e0331153. doi: 10.1371/journal.pone.0331153 (PMC12453241; doi:10.1371/journal.pone.0331153)

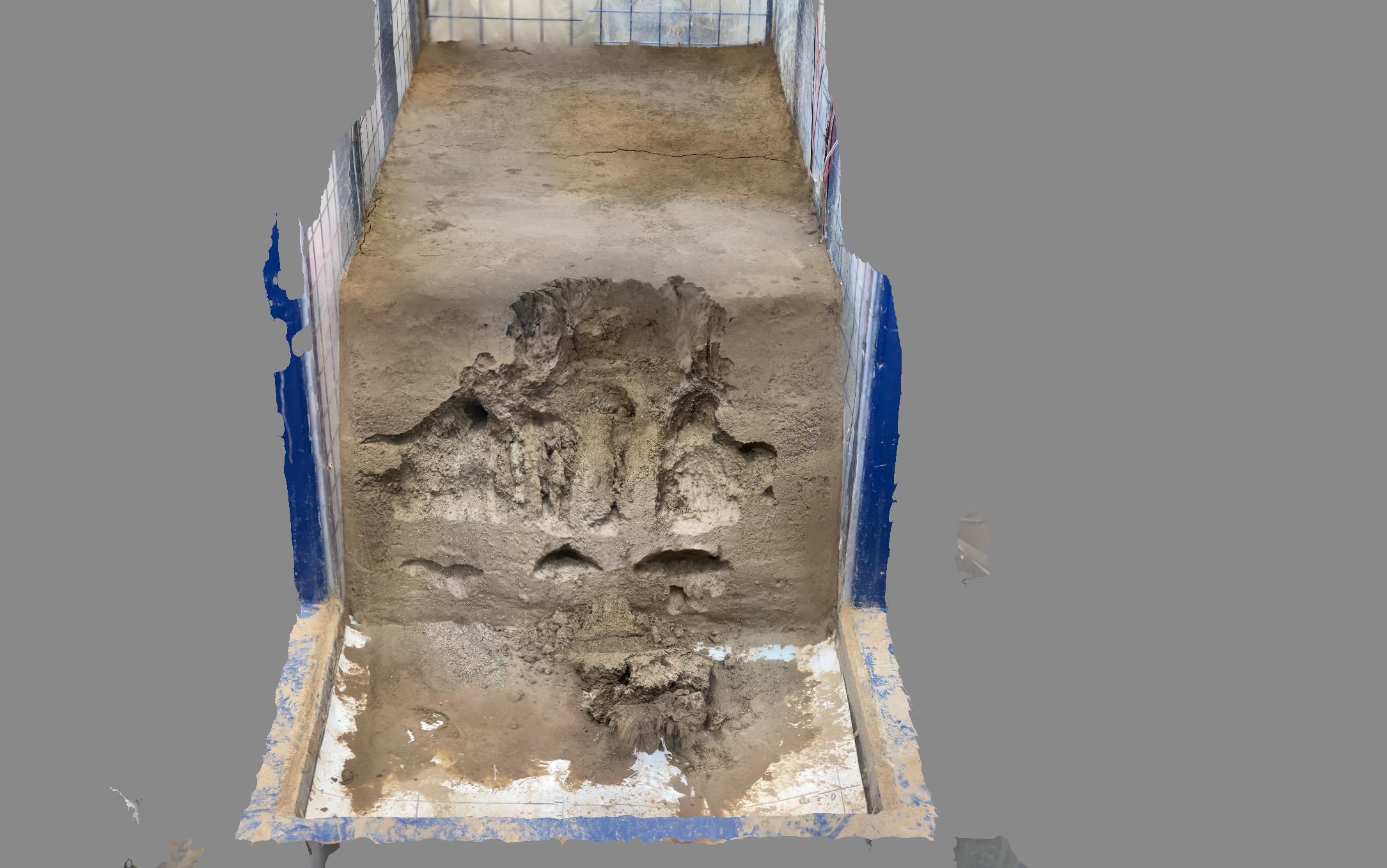

Supplement: S1 File — (ZIP) [file pone.0331153.s001.zip › Fig.7:Layered slope scanning/1.tif]

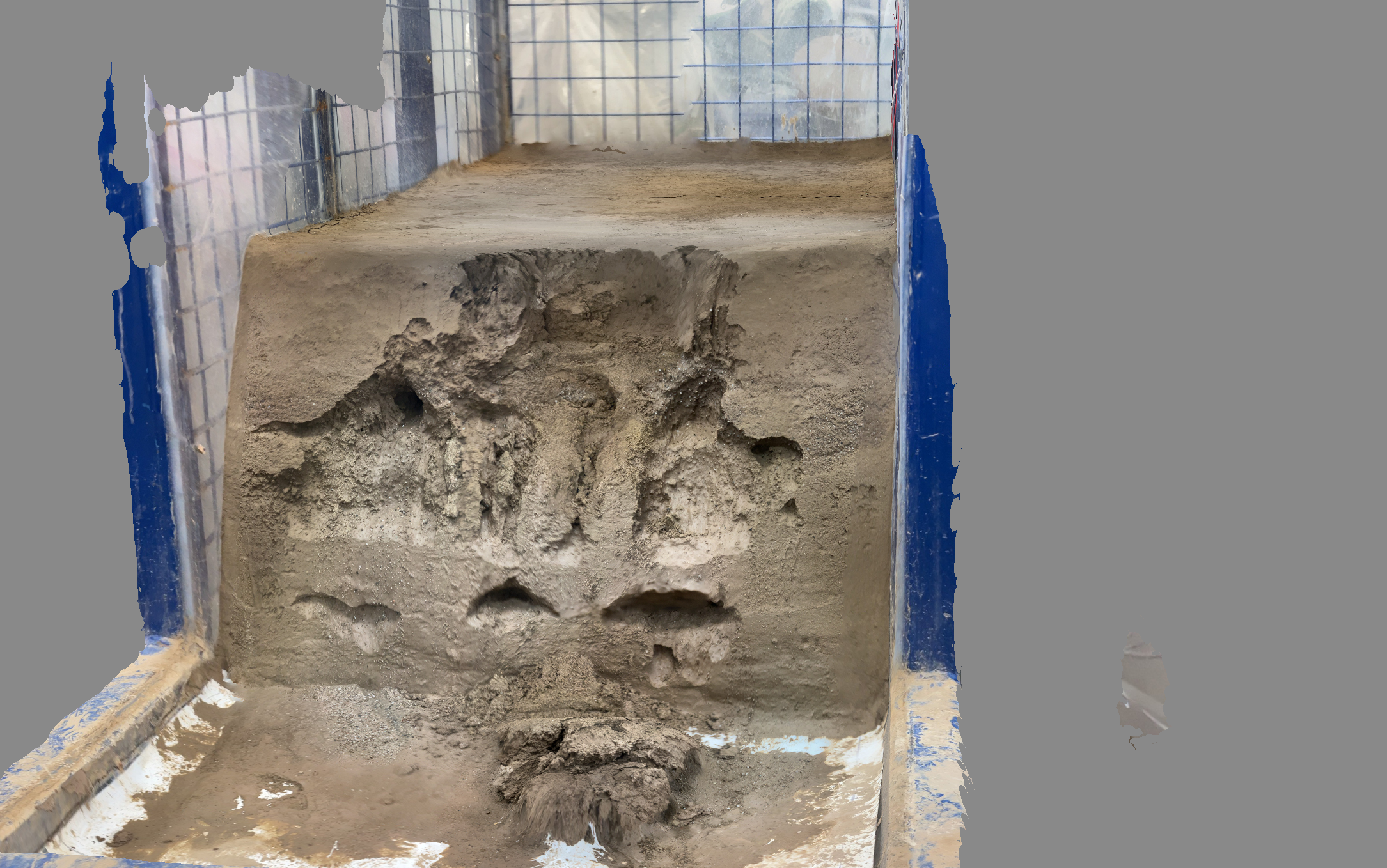

Supplement: S1 File — (ZIP) [file pone.0331153.s001.zip › Fig.7:Layered slope scanning/2.tif]

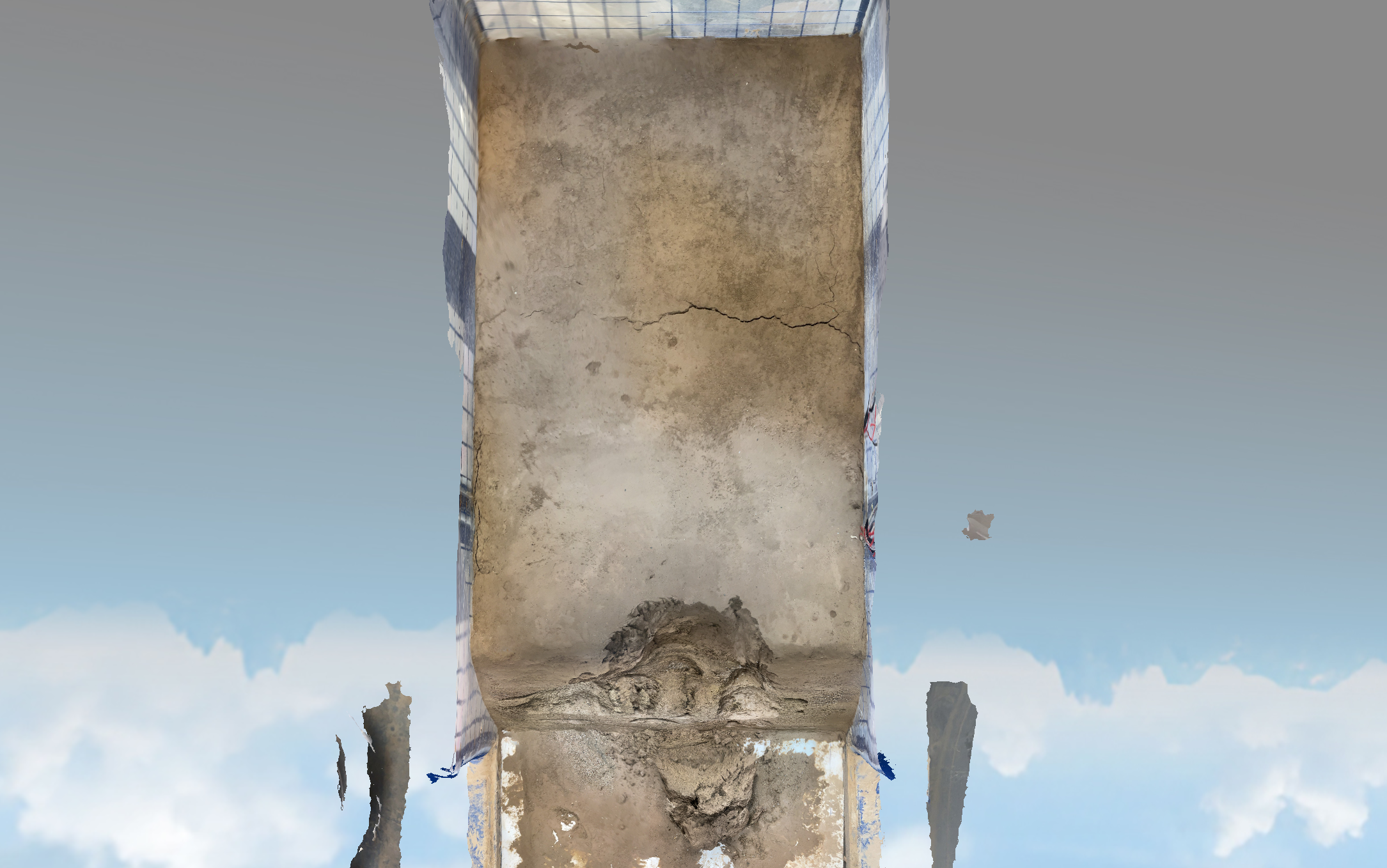

Supplement: S1 File — (ZIP) [file pone.0331153.s001.zip › Fig.7:Layered slope scanning/3.tif]

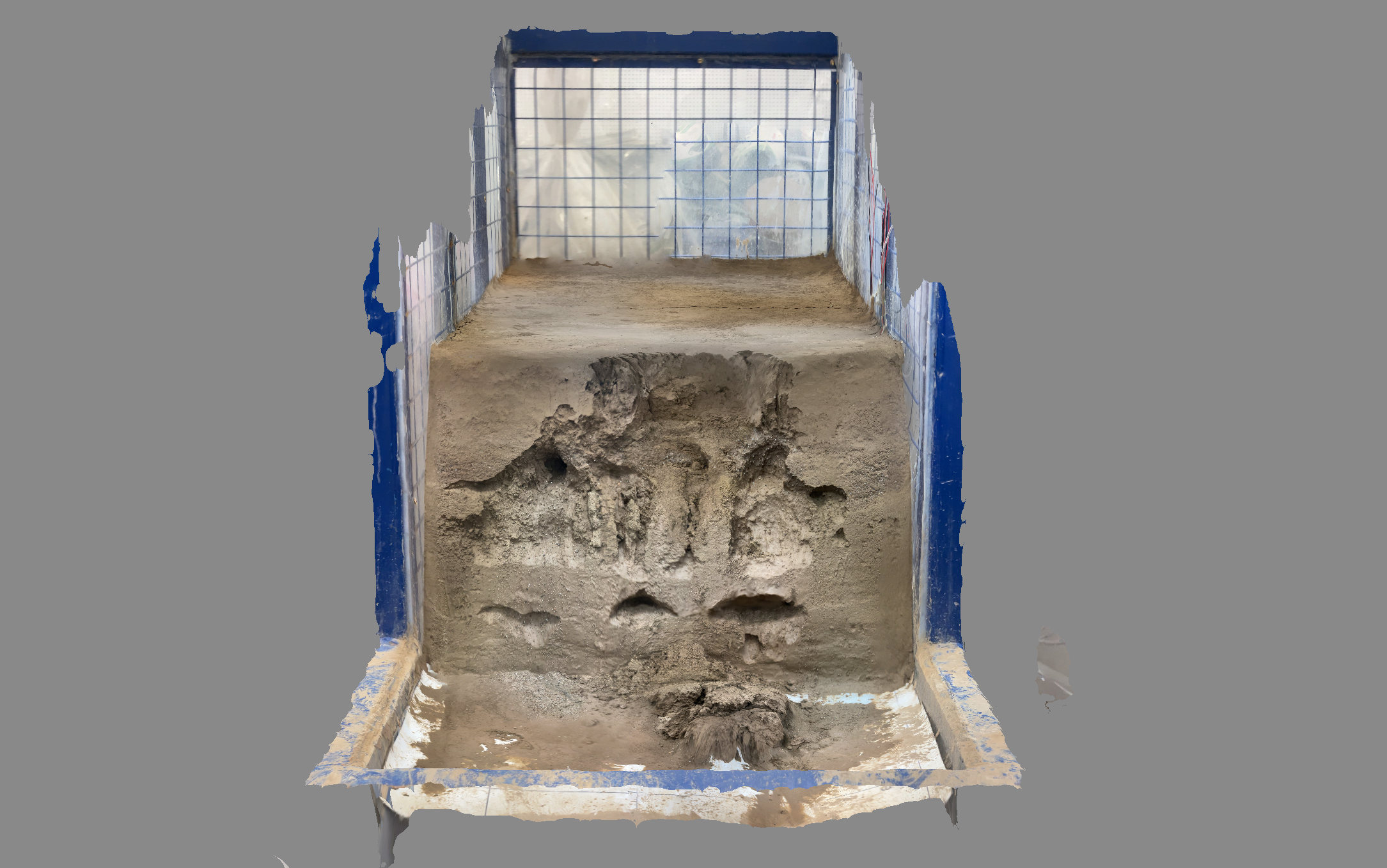

Supplement: S1 File — (ZIP) [file pone.0331153.s001.zip › Fig.7:Layered slope scanning/5.tif]

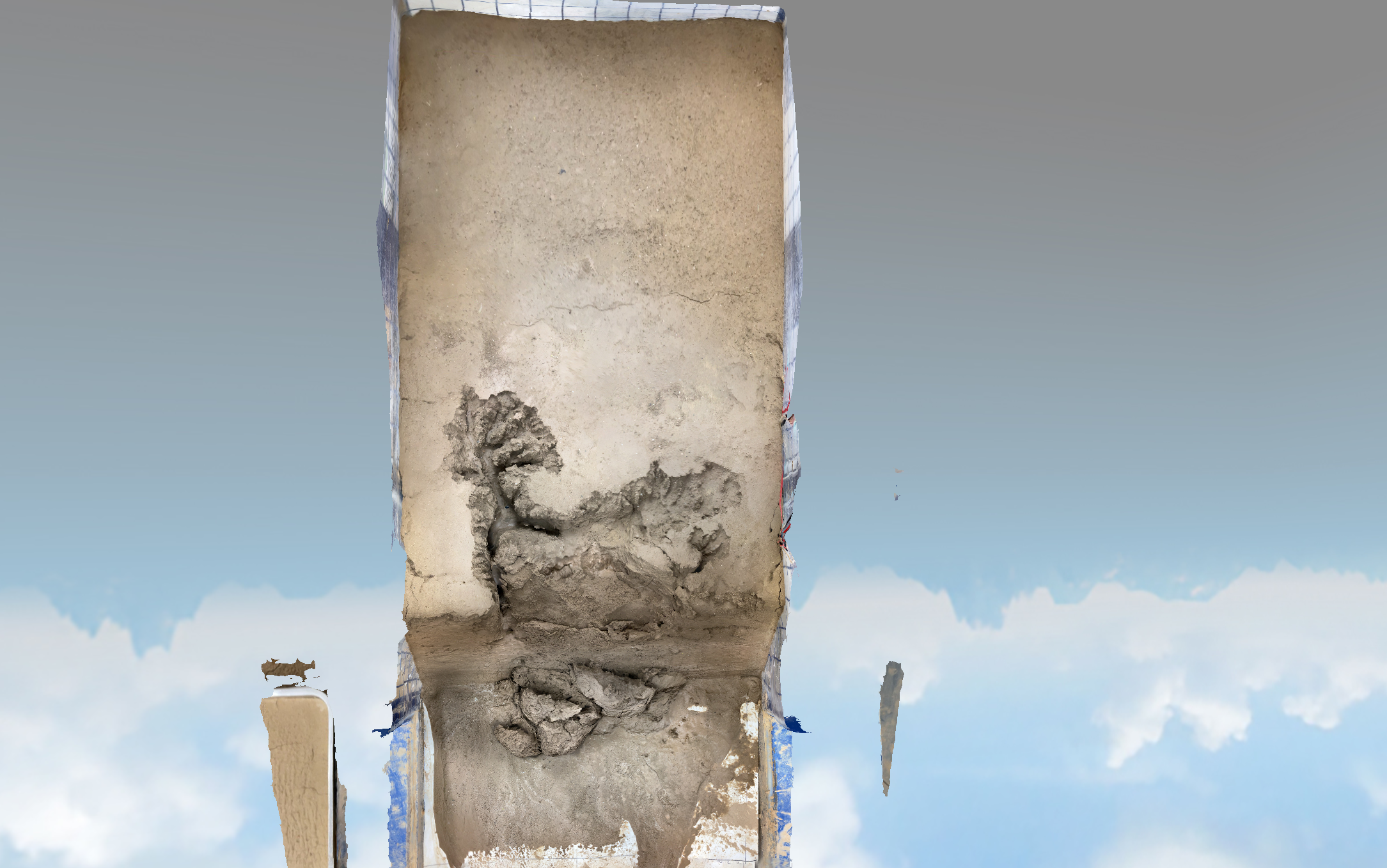

Supplement: S1 File — (ZIP) [file pone.0331153.s001.zip › Fig.7:Mixed soil slope scanning/1.tif]

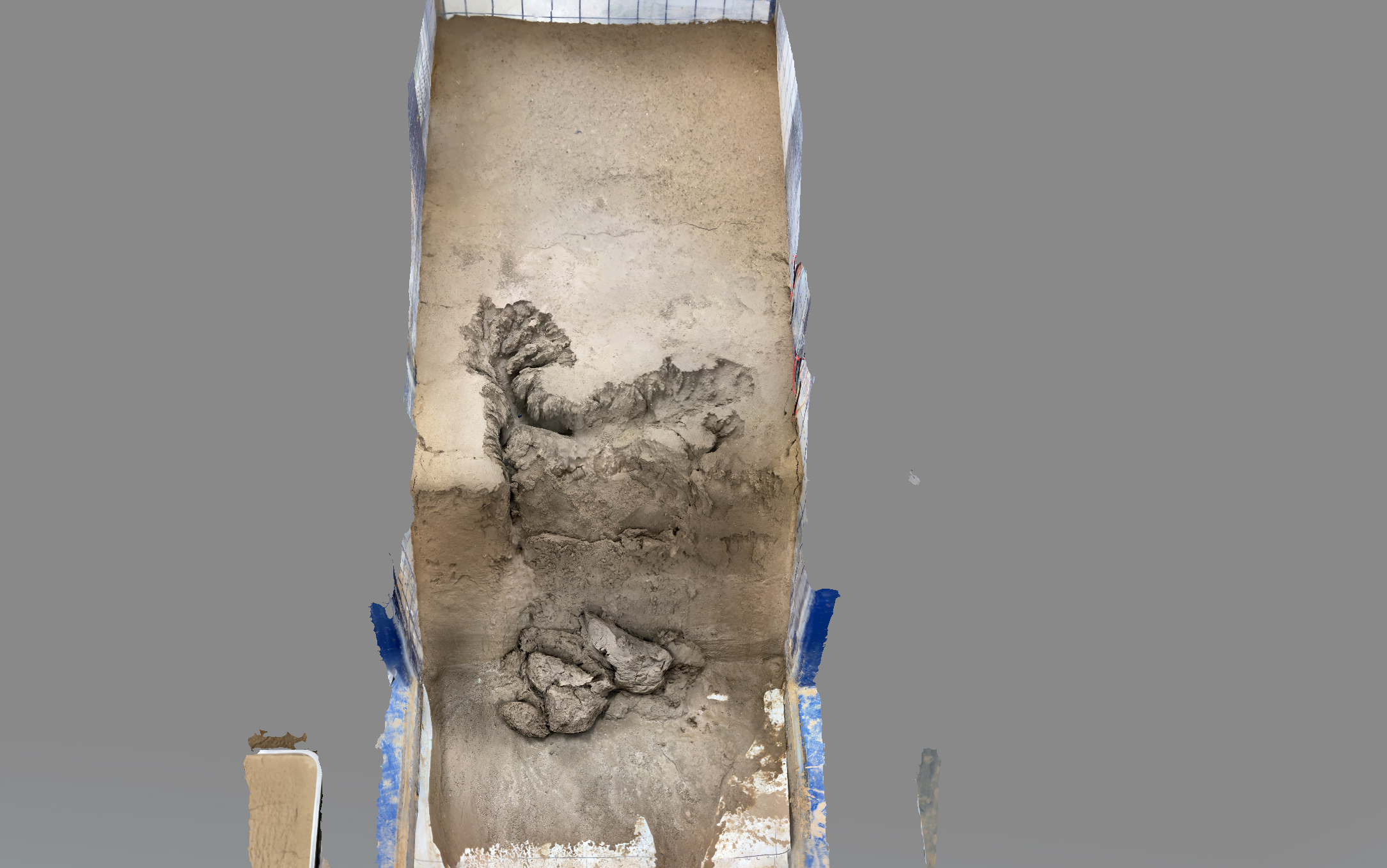

Supplement: S1 File — (ZIP) [file pone.0331153.s001.zip › Fig.7:Mixed soil slope scanning/2.tif]

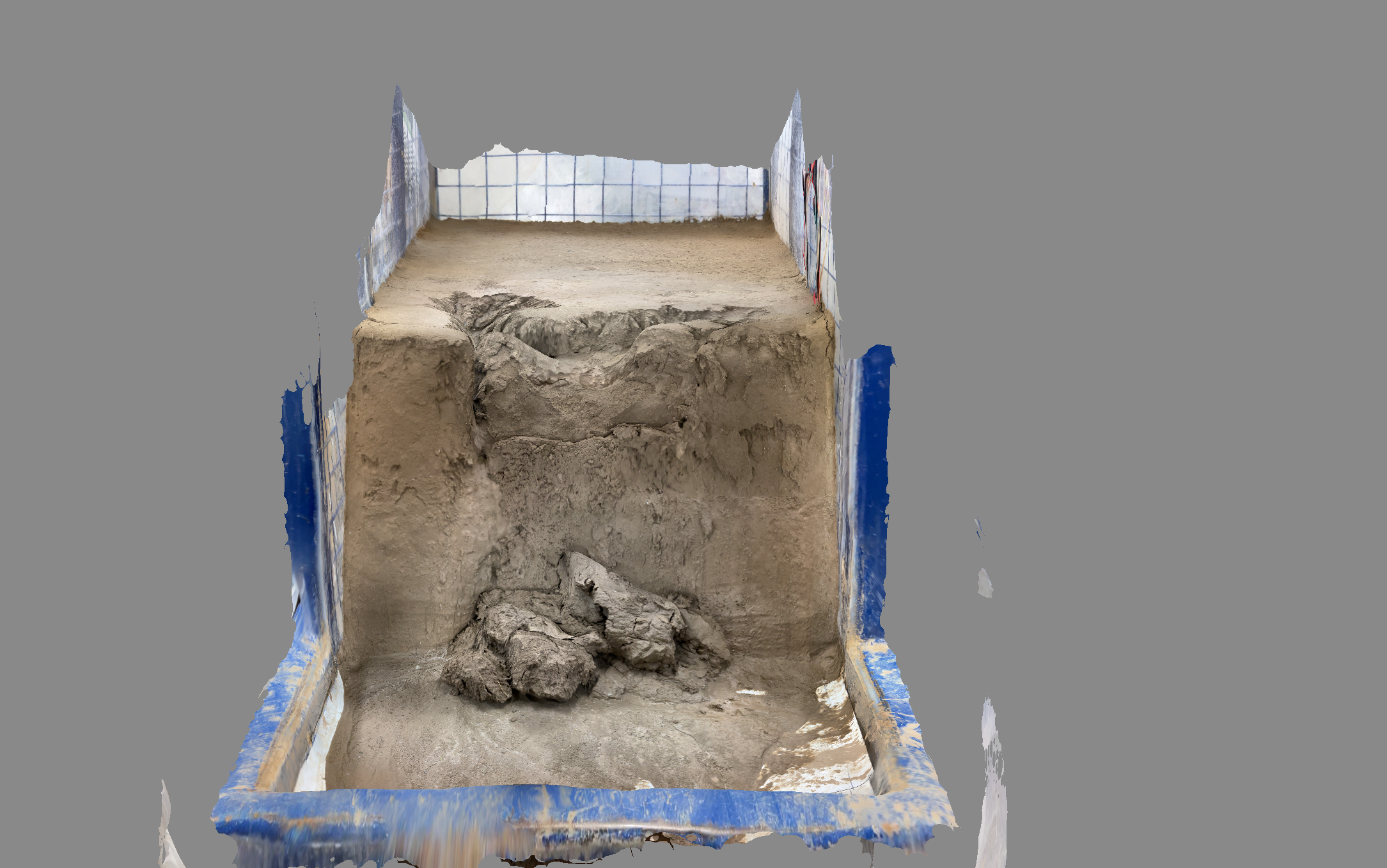

Supplement: S1 File — (ZIP) [file pone.0331153.s001.zip › Fig.7:Mixed soil slope scanning/3.tif]

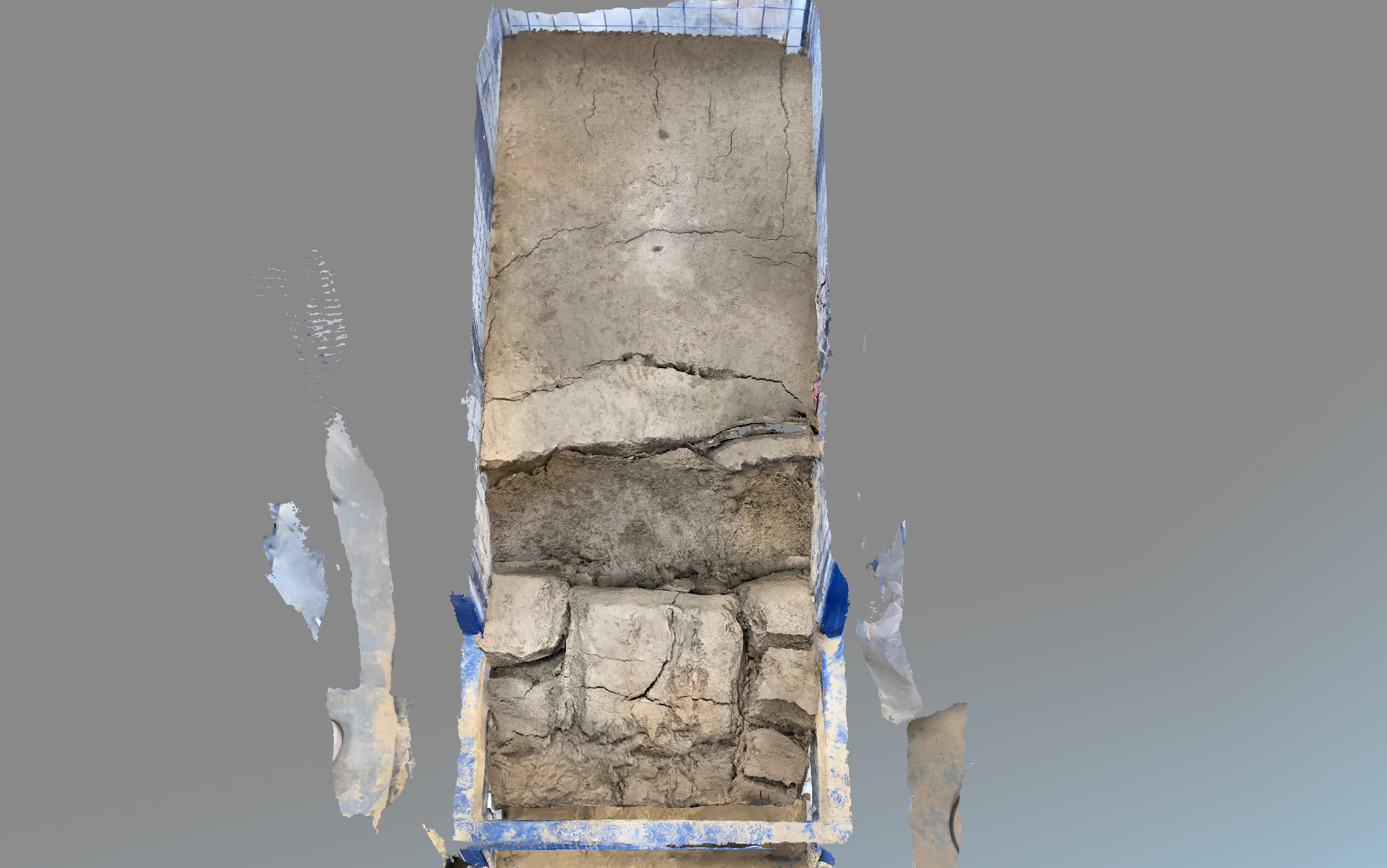

Supplement: S1 File — (ZIP) [file pone.0331153.s001.zip › Fig.7:Scanning of silt slope/1.tif]

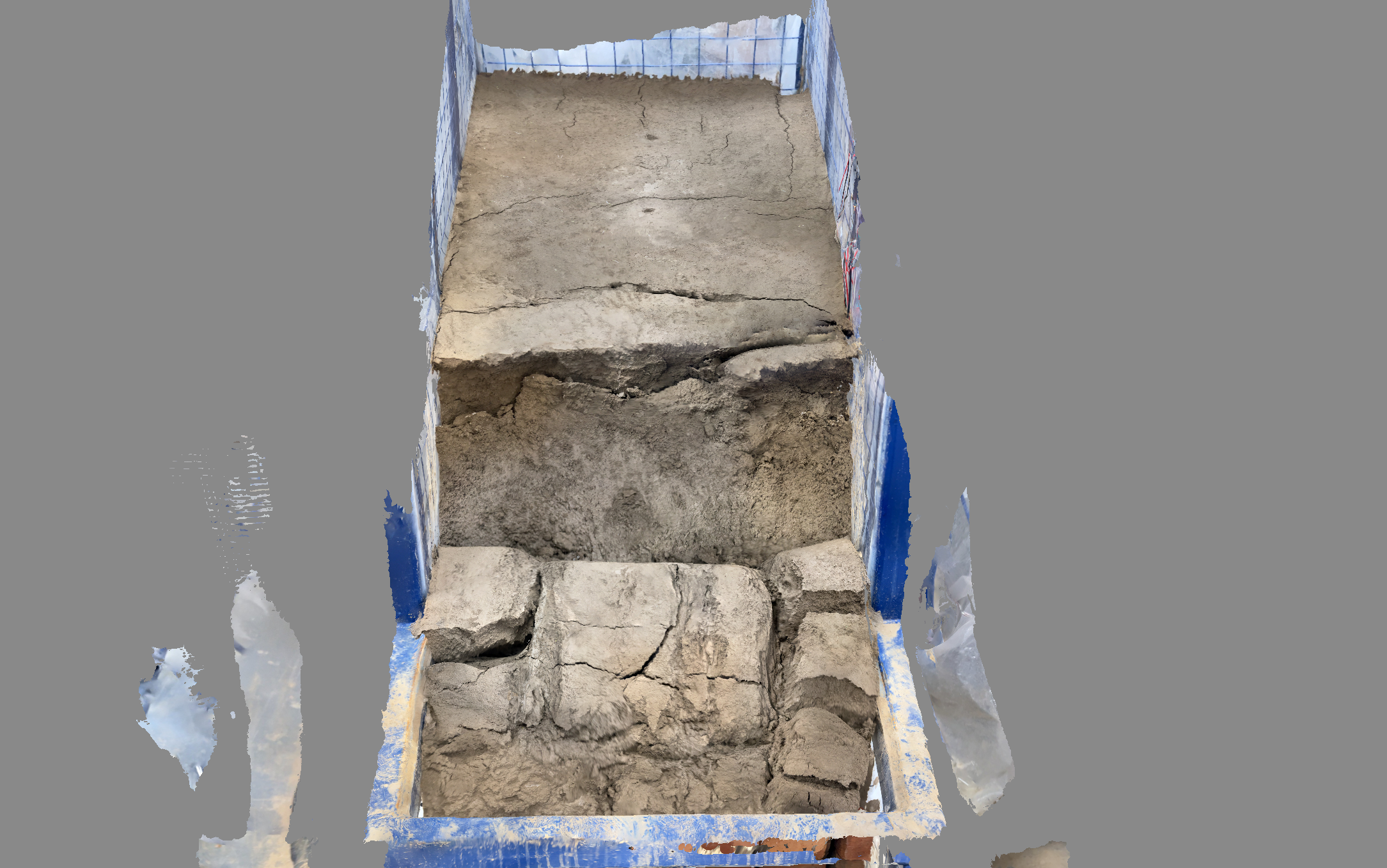

Supplement: S1 File — (ZIP) [file pone.0331153.s001.zip › Fig.7:Scanning of silt slope/2.tif]
